# Expert Opinions and Consensus Recommendations for the Evaluation and Management of Insomnia in Clinical Practice: Joint Statements of Five Italian Scientific Societies

**DOI:** 10.3389/fpsyt.2020.00558

**Published:** 2020-06-26

**Authors:** Laura Palagini, Raffaele Manni, Eugenio Aguglia, Mario Amore, Roberto Brugnoli, Paolo Girardi, Luigi Grassi, Claudio Mencacci, Giuseppe Plazzi, Antonino Minervino, Lino Nobili, Giovanni Biggio

**Affiliations:** ^1^ Psychiatry Division, Department of Clinical and Experimental Medicine, University of Pisa, Pisa, Italy; ^2^ Unit of Sleep Medicine and Epilepsy, IRCCS Mondino Foundation Pavia, Pavia, Italy; ^3^ Department of Experimental and Clinical Medicine, Psychiatric Clinic University Hospital “Gaspare Rodolico”, University of Catania, Catania, Italy; ^4^ Section of Psychiatry, Department of Neuroscience, Rehabilitation, Ophthalmology, Genetics, Maternal and Child Health, University of Genoa, Genoa, Italy; ^5^ IRCCS Ospedale Policlinico San Martino, Genoa, Italy; ^6^ Department of Neuroscience, Mental Health, and Sensory Organs (NESMOS), Faculty of Medicine and Psychology, Sant'Andrea University Hospital, Sapienza University, Rome, Italy; ^7^ Department of Biomedical and Specialty Surgical Sciences, Institute of Psychiatry, University of Ferrara, Ferrara, Italy; ^8^ Department of Neuroscience, ASST Fatebenefratelli Sacco, Milan, Italy; ^9^ Department of Biomedical and Neuromotor Sciences, Alma Mater Studiorum, University of Bologna, Bologna, Italy; ^10^ IRCCS Istituto delle Scienze Neurologiche di Bologna, Bologna, Italy; ^11^ Department of Psychiatry, UOP 25 ASST-Parma, Parma, Italy; ^12^ Department of Neuroscience (DINOGMI), University of Genoa, Genoa, Italy; ^13^ Child Neuropsychiatry, IRCCS Istituto G. Gaslini, Genoa, Italy; ^14^ Department of Life and Environmental Sciences, University of Cagliari, Cagliari, Italy

**Keywords:** insomnia, evaluation, treatment, consensus recommendations, expert opinions, clinical practice

## Abstract

**Background:**

Insomnia is the most commonly reported sleep problem in industrialized countries worldwide being present in about 36.8% of the general population. In Italy, such a percentage seems to be even higher. Although insomnia can be an independent disorder, it is most frequently observed as a comorbid condition and may precipitate, exacerbate, or prolong a broad range of comorbid conditions including physical and mental illnesses. Evaluating and targeting insomnia in the Italian clinical practice should be a priority.

**Methods:**

The present expert options and recommendations development process was based on the RAND/UCLA Appropriateness Method for conceptualizing, designing, and carrying out the appropriateness of procedures for the diagnosis and treatment. Only available options in Italy were taken into considerations.

**Results:**

We evaluated 12 international guidelines and 12 most recent systematic reviews for insomnia evaluation and treatment produced in the last 10 years.

**Conclusions:**

Our findings suggested that symptoms of insomnia must always be assessed in the Italian clinical practice by evaluating nocturnal and daytime symptoms, comorbid conditions and lifestyle. In a patient with chronic insomnia with and without comorbidity, insomnia treatment should be always initiated. CBT-Insomnia therapy should be the first option accordingly to availability. The choice of the drug should be based on different factors such as type of insomnia, age, comorbidities, and potential side effects. Melatonin 2 mg prolonged release should be the first choice in subjects >55 years. If the choice would be a Z-drug or a short-acting benzodiazepine (in subjects <65 years old) or a sedating antidepressant, the use should be in the short term (≤4 weeks) and then proceeds to tapering under clinical monitoring

## Highlights

- Insomnia is the most frequent among sleep and psychiatric disorders.
- Insomnia must always be assessed in the Italian clinical practice.
- Nocturnal, daytime symptoms, comorbid conditions, and patient's lifestyle should be evaluated.
- CBT-Insomnia therapy should be the first option accordingly to availability.
- Pharmacological treatment should be based on different factors such as type of insomnia, age, comorbidities, and potential side effects.
- Melatonin 2 mg prolonged release should be the first choice in subjects ≥55 years (up to 13 weeks).
- Z-drug or a short-acting benzodiazepine (in subjects <65 years old) or a sedating antidepressant, should be used in the short term (≤4 weeks).

## Introduction

Sleep disorders are common in the general population and yet are relatively poorly undiagnosed and untreated despite poor sleep strongly affects the quality of life of many people around the world, including Italy (1–5).

Insomnia is the most commonly reported sleep problem in industrialized countries worldwide and a frequent disorder reported in clinical settings (1, 5–15). An interview, conducted in seven European countries such as France, the United Kingdom, Germany, Italy, Portugal, Spain, and Finland on about 26,000 subjects aged between 15 and 100, showed that insomnia symptoms were very frequent, being present in about 36.8% of the general population (1). In Italy such a percentage seems to be even higher (2, 8, 15). Although insomnia can be an independent disorder, it is most frequently observed as a comorbid condition. In fact it may precipitate, exacerbate, or prolong a broad range of comorbid conditions including physical and mental illnesses (16–22), increasing suicidality and suicide risks (23–25).

Recent studies have demonstrated that interventions for insomnia may favorably impact on the trajectory of psychiatric and medical disorders (26–29).

Since some internationally recommended diagnostic and treatment options along with allotted resources are limited or not available in Italy, the main aim of the paper is to address the problem of the evaluation and treatment of chronic insomnia in the Italian clinical practice, including psychiatric and primary care settings, according to the availability of such options. These recommendations are the results of a working group with an expertise in the field of sleep medicine, psychosomatic medicine, psychiatric practice, and psychopharmacology for responding to the clinical needs emerged from the “real world” practice in the evaluation and treatment of chronic insomnia. Consensus recommendations and joint statements of five Italian scientific societies such as the Italian Association of Sleep Medicine (AIMS), the Italian Association for the Fight Against Stigma (AILAS), the Italian Society of Consultation-Liaison Psychiatry (SIPC), Italian Society of Neuropsychopharmacology (SINF), and the Italian Society of Psychosomatic Medicine (SIMP) are summarized in the paper.

## Treatment choices in the Italian clinical practice

- In a patient with chronic insomnia with and without comorbidity, insomnia treatment should be always initiated in the Italian clinical practice.
- Face-face individual/group therapy or self-help CBT-I should be the first line option accordingly to availability.
- Pharmacological treatment should be first line option when CBT-Insomnia is not available. The choice of the drug should be based on different factors such as type of insomnia, age, comorbidities, and potential side effects among drugs available in Italy:
  Melatonin 2 mg prolonged release (>55 years), sedating antidepressants, short/medium-acting benzodiazepines, Z-drugs
- if the choice is prolonged-release melatonin (>55 years old) use it in within 13 weeks
- if the choice is a Z-drug or a short-acting benzodiazepine (in subjects <65 years old) or a sedating antidepressant, use it in the short term (≤4 weeks) and then proceeds to tapering under clinical monitoring.

## Method

The Italian Society of Psychosomatic Medicine (SIMP) promoted and committed the present expert opinions and consensus recommendations. The development process was based on the RAND/UCLA Appropriateness Method (30) for conceptualizing, designing, and carrying out the appropriateness of procedures for the diagnosis and treatment. Only available options in Italy were taken into considerations (**Table 1**). The method consists of a modified Delphi approach (31) in which a panel of Italian experts assessed the appropriateness of particular clinical decisions to the Italian clinical practice in an iterative way. Initially, a literature review and synthesis were conducted to critically appraise and summarize the evidence (LP, RM). We summarized the main recommendation coming from most recent guidelines about insomnia evaluation and treatment from December 2009 to December 2019, then we performed a literature review on systematic reviews or meth-analyses conducted after the publication of the most recent guideline or conducted during 2017–2019 on the same topic. The PubMed, PsycINFO, and Embase electronic databases were searched for literature published according to the PRISMA (Preferred Reporting Items for Systematic reviews and Meta-Analysis) method (32). Several combinations of search terms were used such as “insomnia evaluation” or “insomnia treatment” and “guideline” or “systematic review 2017–2019” or “meth-analyses 2017–2019”. Inclusion criteria for insomnia evaluation and treatment guidelines were 1) conducted under the umbrella of scientific societies or experts opinion panels, 2) interested adult population, 3) full text available in English or Italian, and 4) performed from December 2009 to December 2019. Inclusion criteria for systematic reviews on insomnia evaluation and treatment conducted across 2017-2019 were 1) interesting adult population, 2) full text available in English or Italian, and 3) conducted according to the PRISMA method (32). In addition, papers were excluded if they interested other sleep disorders or were related to sleep disturbances in general, were conducted in special populations or in children and adolescents who deserve special attention or were limited to oriental medicine. Based on this search, 127 articles were found for insomnia treatment guidelines, 28 for insomnia evaluation guideline, and 29 for insomnia treatment and evaluation systematic review and meth-analyses during 2019. According to the inclusion/exclusion criteria 24 articles were selected: 12 regarded international guidelines on insomnia treatment and evaluation and 12 regarded most recent systematic reviews for insomnia evaluation and treatment produced during 2019.

**Table 1:** Sleep promoting agents suggested for chronic insomnia treatment.

Mechanism of action	Compounds
**GABAa receptor agonists**	**Short/medium half-life benzodiazepines**
	Brotizolam, Lormetazepam, Temazepam, Triazolam
**GABAa1 receptor agonists**	**Z-Drugs** Zolpidem, Zolpidem-PR*, Zaleplon, Zopiclone, Ezopiclone*
**Melatonin receptor agonists**	**Melatonin 2 mg PR** Ramelteon*
**Orexin receptors antagonists**	Suvorexant*
**5HT receptor antagonism**	**Antidepressants**
	Doxepine*, Trazodone

*not available in Italy; PR, prolonged release; GABA, Gamma-aminobutyric acid; 5HT, serotonin.

**Figure 1:**
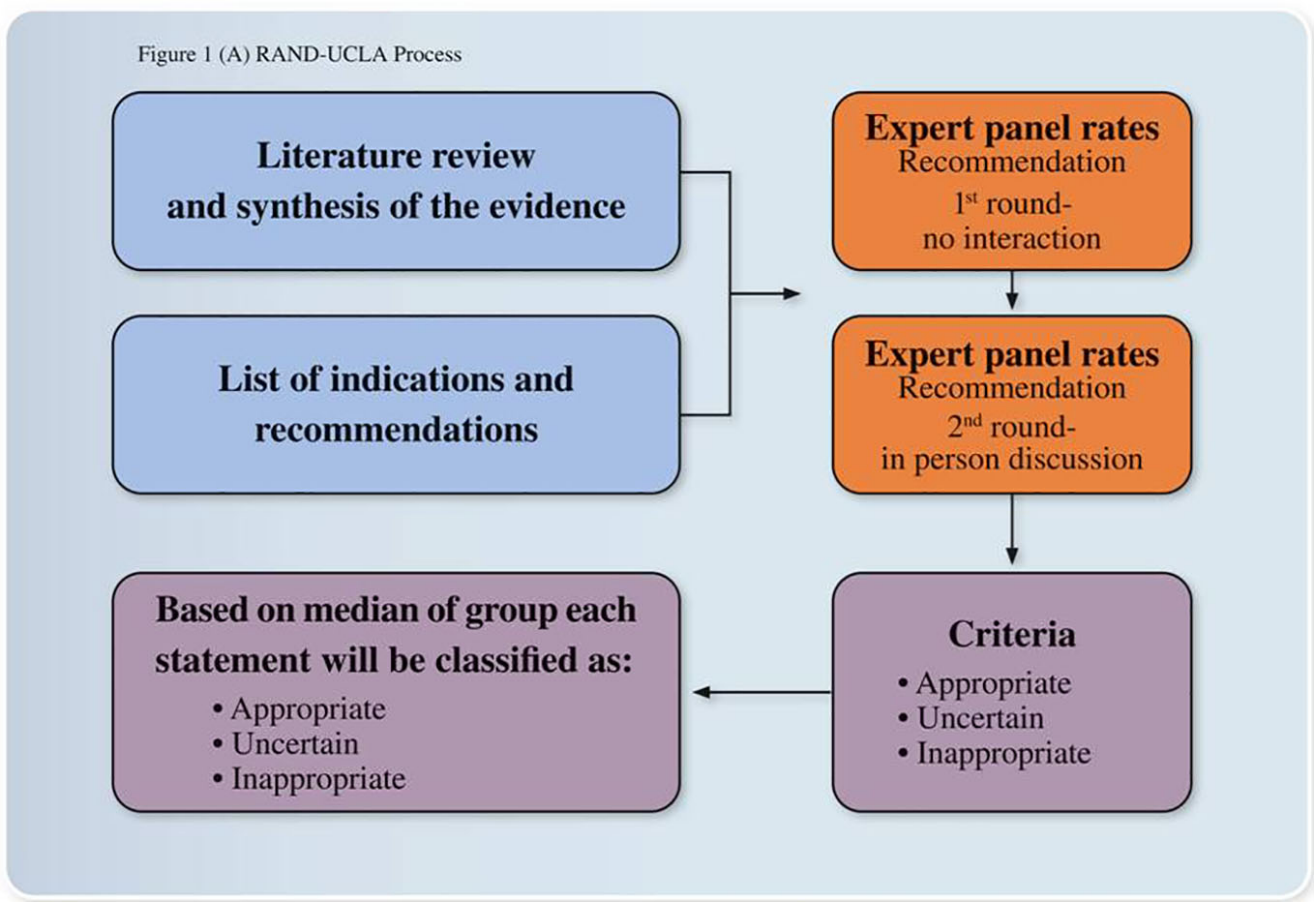
Expert opinions and consensus recommendations process. The development process was based on the RAND/UCLA Appropriateness Method for conceptualizing, designing, and carrying out the appropriateness of procedures for the diagnosis and treatment. The method consists of a modified Delphi approach in which a panel of Italian experts assessed the appropriateness of particular clinical decisions in an iterative way.

The panel of experts in the field was then asked to rate the appropriateness of the recommendations selected according to Delphi approach (31). The Panel experts were asked to rate recommendations in two rounds (1st round with no interaction, 2nd round with an in person discussion) and to express the extent of appropriateness using a 9-point scale in which 9 = extremely appropriate, 1 = extremely inappropriate, and 5 = equivocal or uncertain. Based on the median score and the extent of agreement, for each of the indications, a panel statement was calculated and those statements not achieving consensus were not inserted in the paper. The final Consensus Recommendation Statement resulted from the third round of voting (**Figure 1**). The first part of the paper summarized the main recommendations regarding insomnia evaluation and the second part about insomnia treatment. In this last part, we described the main recommendations of each selected guideline and review by following a chronological order starting from 2009. At the end of each part, we commented in relation to the Italian clinical practice and included our main Recommendation Statements.

### Insomnia

#### Definition and Epidemiology

Chronic insomnia, also currently referred to as “insomnia disorder”, now has similar diagnostic criteria in the American Psychiatric Association's Diagnostic and Statistical Manual of Mental Disorders, Fifth Edition (DSM-5) (12), and in the International Classification of Sleep Disorders (13). Insomnia disorder is now considered a 24-hour sleep-wake disorder (12, 13) characterized by nocturnal and diurnal symptoms.

Approximately 6–10% of the adult population has insomnia that meets the diagnostic criteria (12, 13) while occasional, short-term insomnia affects 30–50% of the population (33). In primary care settings approximately 10–20% of individuals complain of significant insomnia symptoms. There is a higher incidence of insomnia in females, and the incidence increases in both genders with age. Sleep patterns change with normal aging, including advanced sleep timing, shortened nocturnal sleep duration and increased frequency of daytime naps and insomnia symptoms tend to be frequent in people over 65 years. These subjects show more sleep maintenance problems but a decrease in reported daytime problems compared with younger age groups, with little change in prevalence of sleep-onset insomnia. Although insomnia can be a symptom or an independent disorder, it is most frequently observed as a comorbid condition. It may precipitate, exacerbate, or prolong a broad range of comorbid conditions including mental and physical illnesses (16–22). In particular, 40–50% of subjects with insomnia also present a comorbid mental disorder (12), and in this framework, insomnia may impair the trajectory of the disorder being related to its severity, relapses and recurrences, and suicidality (23–25). Moreover, insomnia is related to cognitive impairment (34, 35), it is associated with work disability and reduced work performance (36) and it has a negative impact on the direct and indirect costs of the healthcare system and society (9, 10, 37). Due to its high prevalence, considerable impact on wellbeing, and high medical and society costs, insomnia represents an important healthcare challenge. Despite this, the prevalence of this condition is mostly under-recognized, and many sufferers do not receive adequate treatment (10). Insomnia evaluation and management in early stages should be a priority in order to better identify strategies that improve prevention and treat insomnia and its comorbid conditions.

The presence of insomnia is also frequent in the Italian population. Lugaresi and co-workers conducted the first Italian epidemiological study of insomnia in 1983 in the restricted territory of the Republic of San Marino (6), involving approximately 6,000 subjects out of 20,000 inhabitants. The investigation covered all ages and social classes and revealed that 13.4% of the total population complained of habitually poor sleep. In 1991, an epidemiological survey was carried out on a representative sample of the Italian adult population (38). Fifty-one percent of the approximately 2,000 interviewed subjects reported having experienced insomnia symptoms at least once in the previous year. Transient (one to a few nights) and short-term (1–3 weeks) insomnia had occurred in 15.5% of the population, while 13.2% complained of chronic insomnia. The interview, conducted in the seven above mentioned European countries, had revealed that insomnia symptoms had a prevalence in Italy around 28% (1). In a more recent survey called Studio Morfeo 1, (2, 8) based on a multi-center observational study carried out by general practitioner (GP) homogeneously distributed throughout the Italian national territory and adequately trained at 16 accredited centers for sleep medicine, the prevalence of insomnia was evaluated. Studio Morfeo was carried out to better define these issues in Italy and it was a part of a long-term project sponsored by the Italian Association of Sleep Medicine (AIMS) aimed at improving the understanding among health professionals and the general public of the treatment of sleep disorders. The objective of Studio Morfeo was to determine the frequency of insomnia in a representative population consulting their GP with health problems other than sleep disorders, and to determine the impact of insomnia on the quality of life, health-care resource use, and co-morbidity. A total of 3,284 patients were enrolled by 738 GPs in this Italian survey. Insomnia symptoms were reported by 64% of patients and 44% of them showed a severe insomnia symptomatology. These findings indicate first that insomnia is a frequent disturbance in the Italian primary care population. Secondly, insomnia resulted frequently associated with co-morbid conditions particularly depression and physical illness. Insomnia symptoms also resulted more frequent in females and in the elderly, thus confirming worldwide data about insomnia distribution. Insomnia symptoms resulted making a negative impact on the quality of life and related to an increased use of health-care resources (2, 8). An international survey investigated in 2005 (10) the prevalence and characteristics of insomnia in the general population in Europe, Japan, and the USA through an interview that was conducted over the telephone by professional interviewers. Thirty-seven percent of respondents in Italy, 6.6% in Japan, and 27.1% in the USA reported insomnia, and for many respondents, insomnia was undiagnosed and untreated despite the poor sleep impacting on their daily quality of life. Many individuals who report a complaint of insomnia to their physician leave without a prescription. In particular, only 16% of patients with insomnia were treated by the GP for their sleep problems (2, 8). On the other hand, individuals who do not seek formal care for sleep problems tend to over-medicate their condition by using over-the-counter drugs in inappropriate quantities. In 2018, another study was carried out to evaluate insomnia in an Italian population (15). The study was carried out by GPs. For five consecutive days, each GP was asked to enroll the first patient over 50 years old spontaneously presenting for any medical problems. The Italian version of the Sleep Condition Indicator (SCI) (39) was administered to evaluate insomnia disorder; daytime sleepiness was evaluated by a visual analogic scale. For every patient, GP collected information regarding comorbidities and pharmacological treatment for insomnia and evaluated the severity of insomnia using the Clinical Global Impression Severity Scale. A total of 748 patients (mean age 65, s.d. 9.45) were enrolled by 149 GPs. Prevalence of insomnia disorder was 55.3%. A significant correlation was found among insomnia, sleepiness, and the severity of the patient's illness. The presence of insomnia was more frequently associated with anxiety and depressive disorders or other psychiatric disorders, cardiovascular disease, and chronic pain. Benzodiazepines, z-drugs, antidepressants, and melatonin 2 mg prolonged release were the drugs more frequently used to treat insomnia.

In conclusion, insomnia may affect half of Italian primary care population over 50 years and is frequently associated with different psychiatric and medical conditions, sleepiness, and with a greater severity of the patient's illness.

#### Assessment and Evaluation

Following classification criteria (12, 13) insomnia subjects suffer from both nocturnal and daytime symptoms. Subjects are dissatisfied with sleep quality and quantity and may present one or more of these symptoms: difficulty falling asleep, frequent awakenings, difficulty returning to sleep, and awakening too early in the morning. They also suffer from daytime symptoms such as fatigue, sleepiness, mood disturbance, subjective symptoms of distress, significant distress, or impairment in social, occupational, educational, academic, behavioral, or other important areas of functioning. Insomnia can be episodic lasting for a period ranging from 1 to 3 months or persistent lasting longer than 3 months; transient-episodic forms tend, in the majority of the cases, to chronicity (33).

The evolving models of chronic insomnia according to neurobiological, neurophysiological, cognitive, behavioral, or other perspectives (40–42) made evaluation of insomnia progressively more complex. Although details of current models are beyond the scope of this practice guideline, concepts are critical for insomnia evaluation. The most heuristic model of insomnia is the diathesis-stress model proposed by Spielman et al. (40) more commonly known as the “3-P” model, describing Predisposing, Precipitating, and Perpetuating factors relevant to the development and maintenance of insomnia. *Predisposing factors* include genetic, physiological, or psychological diatheses that confer differential susceptibility to individuals in response to stress. *Precipitating factors* include physiological, environmental, or psychological stressors interacting with predisposing factors to produce acute symptoms. *Perpetuating factors* especially behavioral, cognitive, and environmental factors intervene in the perpetuation of insomnia. Decades of research into the cause of chronic insomnia have identified hyperarousal as a key factor (43) with increased levels of physiological, cognitive, and emotional levels of arousal in insomnia (44). The hyperarousal has been hypothesized to interact with unhelpful cognitive beliefs and negative behaviors contributing to the perpetuation of insomnia. In particular, the identification of perpetuating negative behaviors and cognitive processes often provides the clinician with important information for diagnosis as well as for treatment strategies. In contrast to evolving models and diagnostic classifications for insomnia, procedures for clinical evaluation have remained relatively stable over time. Evaluation continues to need a careful patient history and examination addressing sleep and waking functions as well as common medical, psychiatric, and medication/substance-related comorbidities. International guidelines suggest to evaluate insomnia symptoms firstly using the Consensus Sleep Diary for at least one/two weeks to assess the insomnia day-to-day variability (4, 45, 46). In addition, the administration of questionnaires and survey instruments has been suggested to assesses outcomes and guiding treatment: the Insomnia Severity Index (ISI) (41), the SCI (39) and the Epworth Sleepiness Scale (ESS) (47) are the questionnaires that have been suggested for the evaluation of insomnia and of its daytime consequences (3–5, 45, 46). Both sleep diary and self-reported questionnaires should be collected prior to and during the course of active insomnia treatment and in the case of relapse or reevaluation in the long term (3–5, 45, 46). When a single treatment or combination of treatments have been ineffective a re-evaluation for occult comorbid disorders has been recommended (3–5, 45, 46). The Italian version of the Consensus Sleep Diary (48) of the ISI (49), of the SCI (50), and of the ESS (51) are available. Additional evaluations have been suggested to include the assessment of the predisposing, precipitating, and perpetuating factors (3–5, 45, 46). Particularly for the evaluation of perpetuating negative behaviors and cognitive processes the Dysfunctional Beliefs and Attitudes About Sleep Scale (DBAS) (41) is the most recommended and an Italian version is also available (52).

Both polysomnographic or actigraphic registration are not recommended for the routine evaluation of insomnia. They are suggested if other sleep disorders are reasonably suspected to be related to insomnia. Particularly, polysomnograpy is suggested if sleep-related breathing disorders or sleep-related movement disorders are suspected while the actigraphic registration if disorders of the sleep circadian rhythms are suspected (3–5, 45, 46).

### Recommendation for the Diagnosis and Assessment of Chronic Insomnia in the Italian Clinical Practice

#### First Step

1. Chronic insomnia is a frequent sleep and psychiatric disorder in Italy and worldwide. Symptoms of insomnia must always be assessed in the Italian clinical practice.
2. Insomnia assessment requires to evaluate *nocturnal symptoms* (type, frequency, and duration).
3. An insomnia assessment requires to evaluate *daytime symptoms* (degree of sleepiness and extent of daytime consequences).
4. An insomnia assessment requires to evaluate for the presence of *comorbid conditions* (mental and/or medical and/or another sleep disorder).
5. An insomnia assessment requires to evaluate *daytime lifestyle* of the patient to addressing all the factors that may interfere with sleep (use of substances, pattern of work, school, social life, light exposure, alimentation, exercise, and preferred time for sleeping).

#### Second Step

6) Consider prolonging the clinical interview through the sleep history and detailed medical and psychiatric history.
7) Consider evaluating sleep hygiene behaviors, sleep environment, circadian and predisposing, precipitating and perpetuating factors.
8) Consider using instruments which are helpful in the evaluation of insomnia: a 1/2-week sleep diary to identify general patterns of sleep-wake times and day-to-day variability: the Consensus Sleep Diary (Italian version).
9) Instrumental examination, polysomnography, or actigraphy are not indicated in the routine evaluation of insomnia.
10) If other sleep disorders are reasonably suspected to be related to insomnia, the subjects should be evaluated by a sleep specialist.

### Key Points for the Evaluation of Insomnia in the Italian Clinical Practice

Physicians play a key role in the early phases of insomnia detection and they should evaluate insomnia symptoms in their daily clinical practice. At least insomnia symptoms should be briefly evaluated with a few key points (recommendation 1–5):

1. Problems with sleep during the night: type and duration
2. Feeling of fatigue or sleepiness during the day
3. Medical, mental comorbidities, or other sleep problems
4. Use of caffeine, alcohol, or other substances that may interfere with sleep
5. Daytime lifestyle (work, school, social life, light exposure, alimentation, exercise, and preferred time for sleeping).

### Recommendation for Insomnia Treatment

#### Introduction

According to a mid-90s epidemiological investigation about the use of hypnotics by Italian people (38), 4 million subjects were reported to take sleeping pills, 120 thousands of whom regularly, with most of the subjects taking benzodiazepines to induce or maintain their sleep. A survey about the rate of hypnotic drug prescription in Northern Italy (53) by tracking primary care physicians and psychiatric prescriptions at the pharmacies in five cities, showed the highest prevalence of hypnotic prescriptions in the elderly and women, with the majority of prescriptions being for benzodiazepines, namely lorazepam and triazolam.

Inappropriate benzodiazepine prescriptions were reported in older Italian nursing home residents (54) as well as in the elderly in the general population (55).

To treat insomnia from the onset rather than allowing consolidation and recurrence has been suggested by international guidelines. Although recent international guidelines for the diagnosis and management of insomnia are available (3–5, 46) the herein reported expert recommendations are focused on the Italian context where some drugs are not available. Regarding the therapy of insomnia, while for acute-transient forms pharmacological treatment remain the first line approach (3–5, 46), for chronic insomnia the Cognitive Behavioral Therapy-for Insomnia (CBT-I) is the internationally considered first line treatment (3–5, 46, 56–58) but it is of limited access in Italy. Cognitive behavioral therapy for insomnia usually consists of behavioral strategies including psychoeducation/sleep hygiene, relaxation training, stimulus control therapy, sleep restriction therapy, and cognitive strategies such as sleep/related cognitive restructuring (4). In the context of CBT‐I, psychoeducation typically includes the so‐called “sleep hygiene rules” about health practices and environmental factors (e.g., light, noise, and temperature) that may promote or disrupt sleep. Relaxation therapy is aimed at reducing somatic tension or intrusive thoughts at bedtime. Behavioral strategies, includes sleep restriction and stimulus control therapies; sleep restriction is a method designed to curtail the time in bed to the actual amount of sleep being achieved (40) and Stimulus control therapy is a set of behavioral instructions designed to re‐associate the bed/bedroom with sleep and to re‐establish a consistent sleep-wake schedule (59). Usually, CBT‐I is applied face to face (either on an individual basis or in a group format) by a trained clinician in four‐eight sessions but others forms to deliver the CBT-I have been developed in other countries such as a self-help treatment and a web administered one, and they have been proven to be equally effective (4). However, the availability of CBT-I is to date scarce all over Italy. For this reason, the AIMS, the SIMP, and the Italian Society of Consultation-Liaison Psychiatry (SIPC) are promoting CBT- Insomnia courses all over the country. These courses are conducted by experts in the fields who are first generation CBT-Insomnia therapists and members of the European CBT-Insomnia Academy founded in 2018 under the umbrella of the European Sleep Research Society (ESRS) to widespread CBT-Insomnia therapy all over Europe including Italy. CBT-Insomnia treatment on individual basis or group format applied face to face are available to date only in few sleep centers in Italy but AIMS has started to construct an Italian network of CBT-Insomnia therapists following the European CBT-Insomnia Academy guidelines. A self-help treatment is also available, the Italian version of the “Overcoming Insomnia” “Superare l'insonnia” a self-help guide” (60) is available since 2013, while the web delivery system has not been implemented yet in Italy. Similarly, several pharmacological treatments that are suggested by international guidelines as the pharmacological options for chronic and acute\transient insomnia are not available in Italy.

An Italian consensus report about the evaluation and management of insomnia in general practice was published in 2005 (2). We have now produced the following recommendations for the diagnosis and treatment of insomnia bearing in mind this previous report.

#### Review of Recommendations of the Most Recent International Guidelines and Updated Literature Reviews

Previous international guidelines have recommended that regardless of the type of treatment the need to assess and treat both acute-transient and chronic insomnia forms in the clinical practice should be guided by the grade of impact of this sleep disorder on the patient's sleep quality, health, comorbid conditions, or daytime function. (3–5, 46, 56–58). According to previous international guidelines primary treatment goals in subjects with insomnia should be to improve sleep quality and quantity and to improve insomnia related daytime impairments. Other sleep specific outcome should regards the improvement of wake time after sleep onset (WASO), sleep onset latency (SOL), number of awakenings (NA), total sleep time (TST), or sleep efficiency (SE) (3–5, 46, 56–58).

Summary of consensus statements from the British Association for Psychopharmacology conducted on 2010 and then confirmed on 2019 (3, 5). British Association for Psychopharmacology assessed evidence related to chronic insomnia and defined CBT-Insomnia interventions as the recommended first-line treatment. In addition, the authors conducted a revision about the major drug classes used to treat insomnia in the clinical practice and indicated as first choice for a short-term use Gamma-aminobutyric acid (GABAa) receptor agonists compounds such as short/intermediate benzodiazepines (brotizolam, lormetazepam, temazepam, triazolam) and non-benzodiazepines compounds so called Z-drugs including zolpidem, zaleplon, zopiclone, and ezopiclone. They were found to be effective for the short-term use and to be equally effective in improving SOL, WASO, TST, and SE. In particular, a formulation of zolpidem extended release has shown good results in improving the WASO. A variety of side‐effects have been reported with respects to these classes of drugs including hangover, nocturnal confusion, falls, negative effects on next‐day cognitive performance, rebound insomnia, tolerance, and dependency. On this topic, the authors have suggested, based on systematic reviews available at that time, adverse events/side effects to be less common and less severe for the Z-drugs than short acting benzodiazepines (3, 5) and have suggested to prefer the use of Z-drugs for the short term treatment of insomnia. Indeed, the authors have pointed out that insomnia is often long-lasting and often treated with these hypnotic compounds for long periods in the clinical practice but still the longer-term safety and efficacy of these hypnotics remain uncertain. Trying an intermittent use or to use CBT/Insomnia during tapering was suggested in 2010 and 2019 (3, 5). Commenting about Italy at this point among these compounds ezopiclone, zaleplon, and zolpidem extended release are not currently available.

The authors have also recommended in both 2010 and 2019 melatonin receptor agonists hypnotics as a first-line treatment for insomnia (3, 5). Melatonin production was reported to decline physiologically in middle-aged and elderly subjects and to be lower in patients with insomnia than in good sleepers (3, 5). PRM 2 mg showed to mimic the physiological release of melatonin by releasing melatonin gradually and acting on melatonin receptors. PRM 2 mg has been shown to be effective in improving SOL, WASO, TST, and SE to reduce night awakenings without altering the physiological sleep structure. PRM 2 mg has been shown to e well tolerated and not associated with impairment of psychomotor functions, memory recall, driving skills, and have not shown side‐effects such as hangover, nocturnal confusion and falls, negative effects on next‐day cognitive performance, rebound insomnia, tolerance, and dependency (3, 5, 61). Regarding Italy in the last years a prolonged release melatonin formulation PRM (2 mg), has been approved (also available in Italy since 2013) for the treatment of chronic insomnia characterized by poor quality of sleep in people aged ≥ 55. In Italy, the maximum concentration allowed for melatonin-based food supplements is 1 mg and there is a lack of data that prove the efficacy and safety of the different commercial products. This is of particular importance given the high variability in content compared to the label of melatonin concentrations and the presence of the contaminants as reported in a recent publication by Erland et al. (62). Other compounds which have been shown good efficacy in insomnia treatment in 2010 and 2019 according to the British Association for Psychopharmacology were orexin receptors antagonists but these drugs are not available in Italy and even in Europe. Other compounds such as antidepressants, antihistamines, antipsychotics, or antiepileptics should not be considered first line treatment options for insomnia according to Wilson et al., 2010 and 2019 (3, 5). Indeed, low doses (sub-therapeutic for depression) of sedating tricyclics, particularly amitriptyline, dosulepine, and doxepin, have been used for decades to treat insomnia all over Europe; only doxepine have received the recommendation for insomnia treatment but is not available in Italy. At low dose amitriptyline, is probably the most prescribed antidepressant for insomnia in Italy, is probably acting mostly as a histamine H1 receptor antagonist, although a degree of 5HT2 and cholinergic muscarinic antagonism effect may also contribute to sleep continuity. Anyway, no controlled studies of hypnotic efficacy of low-dose amitriptyline in insomnia have been shown (3, 5). Trimipramine is another tricyclic antidepressant which blocks alpha-1 adrenergic, histamine H1, dopamine D2, serotonin 5HT2, and cholinergic receptors and it is also an antidepressant frequently used in the Italian clinical practice to treat insomnia but few controlled trials have been conducted to prove its efficacy on insomnia (3, 5). Trazodone is an antagonist at 5HT1a, 5HT2, and a1 adrenergic receptors as well as a weak 5HT re-uptake inhibitor; it is the second most prescribed medication for insomnia in the US (46) and its use is very frequent also in Italy (63). In 2010 (3), 18 studies have been conducted with trazodone but only two were conducted in subjects with insomnia and only one was a controlled study. Among SSRI, Paroxetine has shown a good efficacy on sleep, but just in one study and SSRIs, venlafaxine, mianserin, or mirtazapine increases the risk of restless legs syndrome and periodic limb movements in sleep or parasomnias (3, 5). Thus, there was the British Association for Psychopharmacology in 2010 and in 2019 has shown limited evidence for efficacy of doxepin, trimipramine, trazodone, paroxetine in insomnia and recommended to consider the use of antidepressants to treat insomnia when there is a coexistent mood disorder (3, 5). Regarding antipsychotics, olanzapine and quetiapine have shown to improve sleep in healthy volunteers and quetiapine also in subjects with primary insomnia but because the studies were very few and side effects commons with these drugs (weight gain, metabolic syndrome, and extrapyramidal symptoms) no indication was suggested for use of these compounds as first-line treatment in insomnia. Similarly about antihistamines, even if they are frequently used in the clinical practice, in particular in Italy as preparations such as diphenhydramine are available, there were limited evidence of their efficacy on insomnia thus the authors have suggested no indication for use these compounds as first-line treatment in insomnia.

In March 2017, the American Academy of Sleep Medicine wrote the new Clinical Practice Guideline for the Pharmacologic Treatment of Chronic Insomnia in Adults (46). Despite the clearly favorable benefit to risk ratio of CBT-Insomnia, the American Academy of Sleep Medicine (46) underlined that not all patients with insomnia disorder can have access to this treatment or may have benefit from this treatment alone (46). This is the case of Italy where the availability of CBT-I is scarce all over the country. In addition, web administered forms are not available in Italy and although a self-help treatment is available (60) it is scarcely used. Indeed, according to the American Academy of Sleep Medicine (46) CBT-I alone or in combination with pharmacotherapy must continue to be considered as a part of the therapeutic armamentarium for chronic insomnia. Within the suggestions proposed by the American Academy of Sleep Medicine there are many drugs that are not available in Italy such as Ramelteon (melatonin agonist), Ezopiclone and Zolpidem extended release (Z-drugs), and Doxepin (heterocyclic drug). Recommended drugs available also in Italy are Triazolam and Temazepam (BDZ), Zolpidem and Zaleplon, (Z-drugs), Trazodone, and melatonin (as an over the counter preparation). It must be underlined that all these recommendations were *supported by a weak strength of evidence* (46). Considering this last point, it seems that further data are required to formulate any reasonable conclusions and recommendation for insomnia treatment not only for Italy but all over the world (46). As suggested by the American Academy of Sleep Medicine “*clinicians must continue to exercise a reasonable degree of clinical judgment, based not only on the recommendations, but also on clinical experience, prior patient response, patient preferences, and potential adverse effects*” (46).

The European guideline for the diagnosis and treatment of insomnia instead was developed under the umbrella of the ESRS (4) and included the involvement of experts from various European countries including Italy. CBT‐I was recommended as first‐line treatment for chronic insomnia in adults of any age though being the first‐line treatment for insomnia, is not easily available all over Europe. It was assumed that only a minority of patients with chronic insomnia receive this treatment in Europe, including Italy. Thus, the widespread implementation of CBT‐I has been a major challenge for the ESRS thus founding in 2018 the European CBT-I Academy with the aim to widespread this type of therapy. About pharmacotherapy, seven meta‐analyses collected by Reimann et al. (4) have shown that short-medium acting benzodiazepines and Z-drugs are effective in the short‐term treatment (≤4 weeks) of insomnia. Riemann et al. (4) have shown that the newer Z-drugs are equally effective as short acting benzodiazepines on sleep parameters with comparing range of side effects. Long‐term treatment of insomnia with both compounds was indeed not recommended because of a lack of evidence and possible known side‐effects/risks. In patients using these medications on a daily basis, reduction to intermittent dosing was strongly recommended or to tapering during CBT- insomnia treatment confirming indications from Wilson et al. (3, 5) and Satea et al. (46). Ten meta‐analyses were conducted on the efficacy of melatonin (including mainly fast‐release preparations, but also ramelteon and prolonged‐release formulations) in the treatment of insomnia. These meta‐analyses did not provide a uniform picture concerning the efficacy of melatonin and the melatonin receptor agonist ramelteon. Some authors reported that melatonin reduces sleep‐onset latency and improves sleep quality. Some of the original studies also investigated undesired side‐effects and concluded that melatonin is a safe drug. Indeed melatonin was not generally recommended for the treatment of insomnia because of low efficacy even if the grade of this recommendation was weak (4). Four meta‐analyses collected by Reimann et al. (4) have been conducted on the effect of antidepressant on sleep. Only a few randomized controlled trials have evaluated the efficacy of sedating antidepressants. Two meta‐analyses concluded that the efficacy of sedating antidepressants was weaker than that for benzodiazepines and Z-drugs but differently from previous guidelines Riemman et al. (4) suggested to use sedating antidepressant in the short‐term treatment of insomnia including doxepin and trazodone. McCleery et al. (64) described positive effects of trazodone for sleep disorders co‐morbid with Alzheimer's disease. Riemann et al. (4) pointed out that contraindications have to be carefully considered when prescribing antidepressants for insomnia and anyway long‐term treatment of insomnia with sedating antidepressants was not generally recommended because of a lack of evidence and possible side‐effects/risks. Because of insufficient evidence, neuroleptics and antihistaminics were not recommended for insomnia treatment (4).

After these guidelines no other meta-analyses on the topic of hypnotic drugs has been published but two systematic reviews in 2018. One review has shown to also consider sleepwalking as a potential medication adverse effect while treating insomnia that could be more strongly correlated with the use of zolpidem or in particularly with drugs that enhance GABA activity at the GABA_A_ receptor, enhance serotonergic activity, or block the activity of noradrenaline at β receptors. (65). On this topic on April 2019, the USA Federal and Drug Administration-FDA published a Boxed Warning for risk of serious injuries caused by sleepwalking with z-drugs prescription in insomnia. FDA reported data of literature about rare but serious and fatal injuries that have happened with z-drugs because they can cause complex sleep behaviors, including sleepwalking, sleep driving, and engaging in other activities while not fully awake. The other systematic review about the insomnia pharmacotherapy conducted in 2018 concluded that clear pharmacological recommendations for insomnia disorder only exist for the short-term treatment of adults with short acting benzodiazepines and Z-drugs, but in the elderly, benzodiazepines and Z-drugs have advised against, and melatonin might be considered the preferred choice in older patients with insomnia (66).

On the topic of exogenous melatonin, a meta-analysis has shown the efficacy in reducing SOL in primary insomnia (67). Four systematic reviews published in 2018 have supported these evidence (66, 68–71) and another meta-analysis conducted in 2019 confirmed the efficacy of melatonin on SOL and sleep quality (71). These reviews pointed out the efficacy of prolonged-release melatonin 2 mg in subjects older than 55 years when levels of melatonin start to decrease (68, 69). In particular, Quera-Salva et al., (69) highlighted clinically relevant benefits of prolonged-release melatonin 2 mg in sleep quality and latency, next-day morning alertness and quality of life. The authors have shown that an oral 2 mg dose of prolonged release melatonin once daily, for 3 months, is generally well tolerated with no rebound, withdrawal or ‘hangover' effects and no safety concerns on concomitant therapy with antihypertensive, antidiabetic, lipid-lowering, or anti-inflammatory drugs. Untoward effects of hypnotics on cognition, memory, postural stability, and sleep structure were not seen with prolonged-release melatonin 2 mg and its effects on sleep and daytime parameters were maintained or enhanced over the 6-month period with no signs of tolerance. Given as a first-line prescription, through the 13 weeks' posology and the lack of rebound effects, prolonged-release melatonin 2 mg has shown the potentiality to improve quality of life in insomnia patients aged 55 years and older and avoid long-term use of hypnotics (3, 5, 69).

No other meta-analyses on the topic of antidepressants has been published after these guidelines were produced, but two systematic reviews confirming the evidence of a small improvement in sleep quality with the short‐term use of low‐dose of trazodone compared with placebo, while no evidence emerged for amitriptyline despite common use in the clinical practice for insomnia (72). The blocking of the 5-HT2A, histamine H1, and alpha receptors is thought to produce the hypnotic effect reported for low doses of trazodone (25–100 mg). At these low doses, trazodone induces and maintains sleep without causing daytime drowsiness or tolerance, mainly because of its short half-life (3–6 hours) (73).

There were no meta‐analyses on the efficacy of antipsychotics in insomnia, but four related systematic reviews have been conducted and concluded that because of insufficient evidence and in light of their side‐effects, antipsychotics are not recommended for insomnia treatment in the absence of psychiatric disorders. A systematic review on the effect of second-generation antipsychotics supported the evidence that second generation antipsychotics including clozapine, olanzapine and paliperidone may ameliorate insomnia but in the context of schizophrenia (74). One of these systematic review about the insomnia pharmacotherapy conducted in 2018 concluded that clear pharmacological recommendations for insomnia disorder only exist for the short-term treatment of adults with short-acting benzodiazepines, Z-drugs, and sedating antidepressants trazodone, if CBT-I is not available or not effective. In the elderly with insomnia, benzodiazepines and Z-drugs are advised against, while prolonged release melatonin 2 mg might be the preferred choice (66) and noteworthy that add-on of prolonged released melatonin 2 mg to standard therapy has positive effects on cognitive functioning and sleep maintenance also in Alzheimer Disease compensated patients with insomnia compared to placebo.

**Figure 2:**
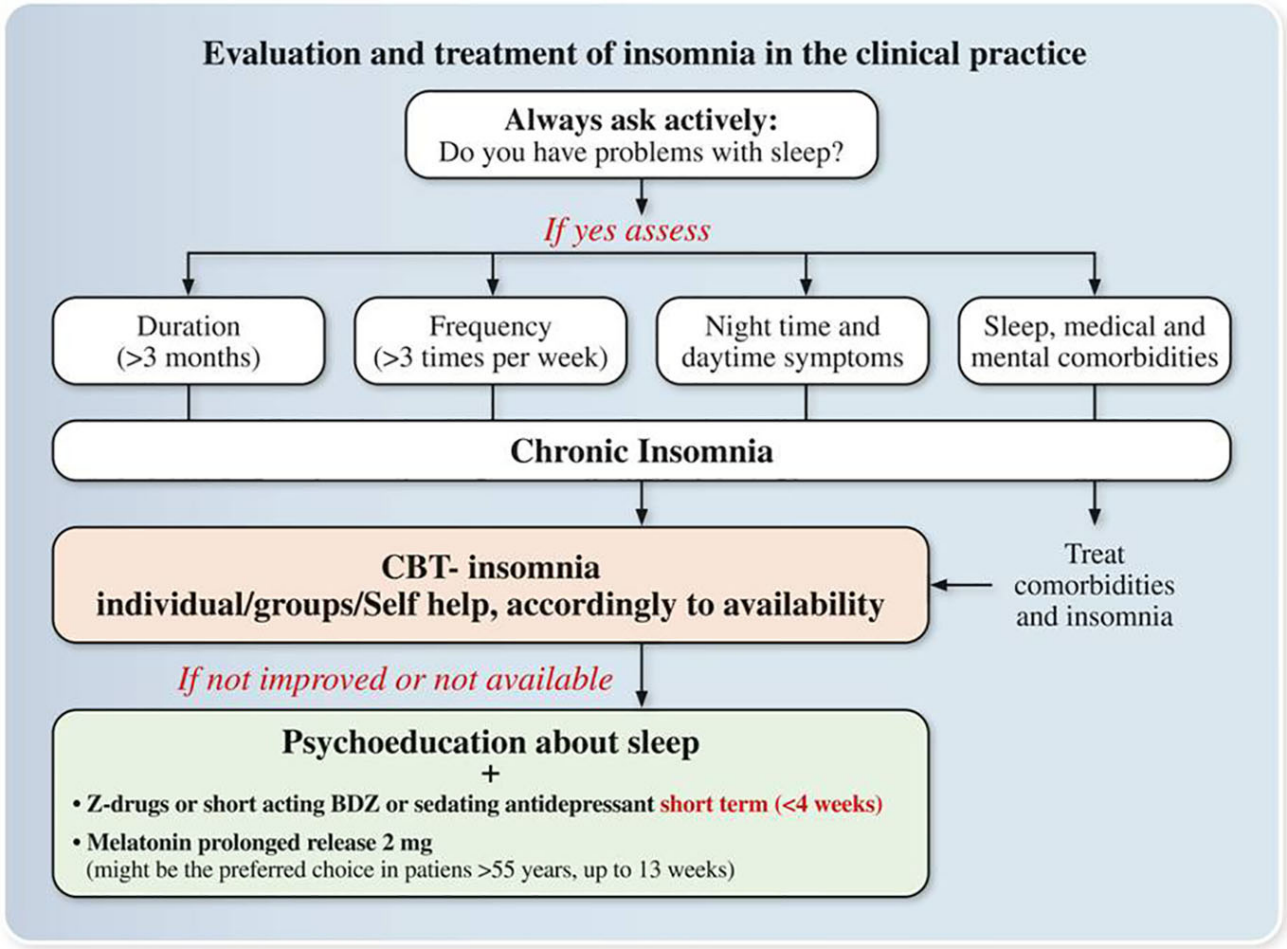
Expert opinions and consensus recommendations on the Evaluation and treatment of insomnia in the clinical practice. Z-drugs: Zolpidem, Zaleplon, and Zopiclone; BDZ: benzodiazepines.

Concluding a review of current literature suggested as a first line option for the treatment of chronic insomnia CBT-Insomnia. The availability of CBT-Insomnia is to date scarce all over Italy, but it is available in sleep centers in Bologna, Milan, Pisa, and Rome. In addition, AIMS, SIMP, and SIPC are promoting CBT-Insomnia courses all over the country in accordance with Italian first-generation CBT-Insomnia therapists who are members of European CBT-Insomnia Academy (including LP) (75). Hence, hopefully in the next few years CBT-Insomnia availability would be improved all over the country. Since a self-help treatment is available in Italian language (60), this may help in currently delivering CBT-Insomnia all over Italy. Regarding pharmacological therapies GABA receptor agonists compounds such as short/intermediate benzodiazepines brotizolam, lormetazepam, temazepam, triazolam are available in Italy and the Z-drug zolpidem is also available. Literature support their short-term use (< 4 weeks) in adults, but in the elderly, benzodiazepines and Z-drugs have advised against in light of their side‐effects. Among agonists of melatonin receptors exogenous Melatonin 2 mg prolonged release is the only compound available in Italy and it should be the preferred choice in subjects >55 years in light of its efficacy and limited side‐effects can be used within 13 weeks. In the last few years, it is emerging the role of sedating antidepressants for the treatment of insomnia. To date Trazodone is the only compound available in Italy which has shown some potentiality at low doses, but it should be used in the short term (<4 weeks). Other compounds are not available in Italy such as orexin antagonists or are not suggested for the treatment of chronic insomnia such as neuroleptics or antihistaminics (**Figure 2**).

#### Insomnia Treatment Goals/Treatment Outcomes: Recommendations for the Italian Clinical Practice

Regardless of the therapy, primary treatment goals for chronic insomnia should be:

1. to improve sleep quality and quantity
2. to improve insomnia related daytime impairments.

Treatment choices:

- In a patient with chronic insomnia with and without comorbidity, chronic insomnia treatment should be always initiated in the Italian clinical practice.
- Face-face individual/group therapy or self-help CBT-I should be the first line option accordingly to availability.
- Pharmacological treatment should be first line option when CBT-Insomnia is not available. The choice of the drug should be based on different factors such as type of insomnia, age, comorbidities, and potential side effects among those drugs available in Italy:
  Melatonin 2 mg prolonged release (>55 years), sedating antidepressants, short/medium-acting benzodiazepines, Z-drugs
- if the choice is prolonged-release melatonin (>55 years old) use it in within 13 weeks
- if the choice is a Z-drug or a short-acting benzodiazepine (in subjects <65 years old) or a sedating antidepressant, use it in the short term (≤4 weeks) and then proceeds to tapering under clinical monitoring.

## Author Contributions 

LN, LP, and RM managed the literature search and finalized the article. All authors contributed to the article and approved the submitted version.

## Conflict of Interest

The authors declare that the research was conducted in the absence of any commercial or financial relationships that could be construed as a potential conflict of interest.
